# In Silico Characterization of Sirtuins in Acetic Acid Bacteria Reveals a Novel Phylogenetically Distinctive Group

**DOI:** 10.3390/molecules30030635

**Published:** 2025-01-31

**Authors:** Igor Jugović, Janja Trček

**Affiliations:** 1 Department of Biology, Faculty of Natural Sciences and Mathematics, University of Maribor, SI-2000 Maribor, Slovenia; igorjugovic444@gmail.com; 2 Department of Microbiology, Biotechnical Faculty, University of Ljubljana, SI-1000 Ljubljana, Slovenia

**Keywords:** acetic acid bacteria, *Acetobacter*, *Novacetimonas*, aging, sirtuins, SirAAB, SirAAB-L, SirAAB-S

## Abstract

Acetic acid bacteria are single-celled organisms well-known for their ability to convert ethanol into acetic acid. Still, recent research suggests they may harbor another attractive characteristic—the production of proteins with remarkable similarities to sirtuins. Sirtuins have been linked to lifespan extension in various organisms, raising intriguing questions about the potential connection between acetic acid bacteria and the biology of aging. This article delves into the characterization of sirtuin homologs in acetic acid bacteria. Up to three types of sirtuin homologs have been identified in 21% of acetic acid bacteria genomes deposited in NCBI. All three types were present only in the genera *Acetobacter* and *Novacetimonas*, which are known to survive in the harshest environmental conditions (high concentrations of acetic acid and ethanol). Interestingly, two types of these sirtuin homologs (SirAAB-L and SirAAB-S) constitute a separate group (SirAAB), distinctive from all other presently known sirtuins. The results obtained in silico thus encourage further studies into the function of these types of sirtuins and their interplay with metabolic pathways in these industrially important bacteria.

## 1. Introduction

Sirtuins, also known as the SIR2 family of proteins, are a class of NAD^+^-dependent enzymes. The “sirtuin” name is derived from the yeast gene “silent mating-type information regulation 2 (SIR2)”, the gene responsible for cellular regulation in yeast [1,2].

**Figure 1:**
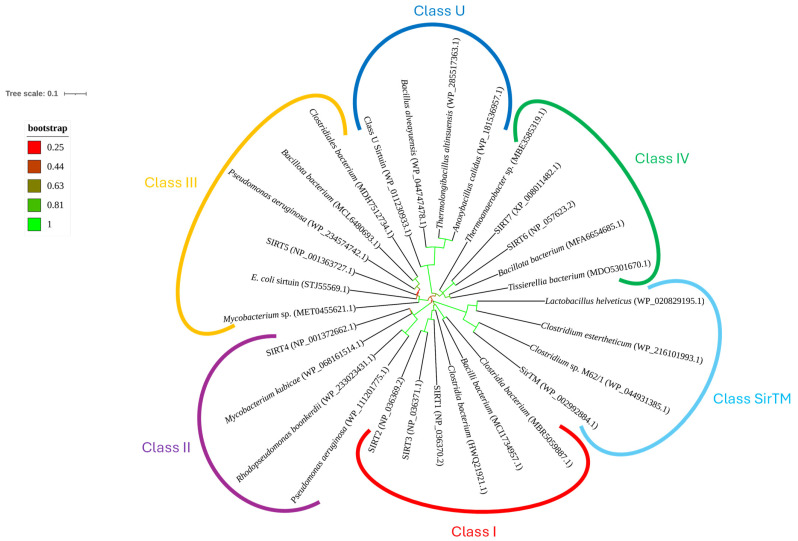
Unrooted phylogram illustrating the relationship between the known sirtuin classes (I–IV, U, and sirtuins associated with macrodomains (SirTM)). Sirtuins labeled from SIRT1 to SIRT7, Class U sirtuin, *Escherichia coli* sirtuin, and SirTM represent the reference sequences, which are surrounded by other sequences from various bacterial species. SIRT1-SIRT7 represent human sirtuins, Class U sirtuin is represented by *Geobacillus* sp. and the SirTM sirtuin by *Streptococcus* sp. Class U and Class SirTM are more distinct from the other classes. The evolutionary history was inferred using the Neighbor-Joining method [6]. The optimal tree is shown. The tree is drawn next to the tree scale (upper left corner), with branch lengths in the same units as those of the evolutionary distances used to infer the phylogenetic tree. The scale length is 0.1, which corresponds to 10 substitutions per 100 positions or 10% sequence divergence in the phylogenetic tree. The bootstrap values (ranging from 0 to 1) are presented by colored branches, with green branches having the highest bootstrap values and red branches having the lowest bootstrap values. A legend for bootstrap values is provided under the tree scale. The evolutionary distances were computed using the p-distance method and are in the units of the number of amino acid differences per sequence. The rate variation among sites was modeled with a gamma distribution (shape parameter = 1). This analysis involved 29 amino acid sequences. All ambiguous positions were removed for each sequence pair (pairwise deletion option). There were a total of 377 positions in the final dataset.

The classification of sirtuins began in 2000 with Frye’s pioneering work. By comparing the sequences of the conserved sirtuin core domain (which includes the NAD^+^-binding Rossmann-fold motifs), Frye identified four main clusters, subsequently named Class I, II, III, and IV. This classification was based on distinct sequence motifs and branching patterns in phylogenetic trees, reflecting evolutionary divergence [3]. After the initial Classes I–IV were described, researchers found additional sirtuins that did not cluster robustly into any of the four major lineages. These sirtuins often appeared in Gram-positive bacteria, but their phylogenetic positions were unsolved; they do not confidently nest in Classes I–IV. Because of this, the scientists began referring to them collectively as Class “U”, short for “undetermined” [4]. As genomic data expanded, especially from extremophilic bacteria and archaea, another distinct cluster of sirtuins emerged, prominently including an enzyme from the hyperthermophilic bacterium *Thermotoga maritima*. Because these sirtuins formed a discrete branch separate from Classes I–IV and from Class U (Figure 1), researchers designated them as SirTM, standing for “Sirtuin from *Thermotoga maritima*”. Structural and sequence analyses showed unique features in the catalytic domain of *T. maritima* sirtuins that suggest a separate lineage [5].

Furthermore, each class has its own distinct though overlapping biochemical characteristics and substrate preferences.

Class I consists of sirtuins found in eukaryotes (fungi, plants, and animals) and some prokaryotes. It includes *Saccharomyces cerevisiae* Sir2 (the founding sirtuin) and human SIRT1, SIRT2, and SIRT3. They predominantly catalyze NAD^+^-dependent deacetylation of histone proteins (e.g., H3K9 and H4K16), transcription factors (e.g., p53, FOXO, and NF-κB), and metabolic enzymes (e.g., acetyl-CoA synthetase 2, and glutamate dehydrogenase), but some can also catalyze ADP-ribosylation under certain conditions. Thus, they play an important role in regulating chromatin structure gene expression, stress response, metabolism, and cell survival [1,3,7].

In Class II, there are sirtuins found in certain fungi (e.g., *S. cerevisiae* Hst4) and some bacteria. They conduct NAD^+^-dependent deacetylation with modest substrate specificity (histones and non-histone proteins) differences from Class I. They are particularly active under stress conditions or during specific cell cycle phases [1,3,7].

Class III sirtuins are abundant in bacteria (e.g., CobB in *E. coli*) and some eukaryotes. While their primary reaction is NAD^+^-dependent deacetylation, some members exhibit desuccinylation or demalonylation in bacteria and archaea. They deacetylate critical enzymes, histones, transcription factors, or stress-response proteins, thus modulating central metabolism as well as regulating DNA repair and gene expression [3,8].

Class IV sirtuins are found in archaea and thermophilic bacteria. They are also identified in some eukaryotic lineages, though less common. They predominantly catalyze NAD^+^-dependent deacetylation, potentially with additional ADP-ribosylation activities, and modify histone-like proteins in archaea and metabolic and stress-related proteins in hyperthermophiles. With this, they help organisms adapt to extreme environments (e.g., high temperature, low pH), maintaining protein integrity and regulation under stress [3,8].

Class U is a collective group of undetermined sirtuins exclusively found in Gram-positive bacteria. Through NAD^+^-dependent deacetylation, they deacetylase different metabolic enzymes and stress-response factors, consequently playing an essential role in stress responses and metabolic regulation, particularly under oxidative stress and nutrient scarcity conditions [5].

As for the Class SirTM sirtuins, they are found in pathogenic bacterial and fungal species as well as in hyperthermophilic bacteria. They are also known as macrodomain-linked sirtuins. Although their unique functional roles are not yet fully understood, they are often linked to virulence and survival in hostile environments [5].

In summary, bacterial sirtuins play a vital role in regulating metabolic pathways that enable these organisms to survive under challenging conditions, such as high acidity or osmotic stress [8]. They directly or indirectly contribute to the modulation of enzymes involved in the TCA cycle, gluconeogenesis, and lipid metabolism, promoting energy efficiency and providing protection against oxidative damage [9,10]. These metabolic pathways are also important in acetic acid bacteria (AAB) [11,12,13], indicating that their sirtuins may play similar roles in enzyme modulation as observed in other bacteria [8]. This connection between sirtuins and metabolic regulation in bacteria reflects the ancient evolutionary origins of these proteins and their conserved role in cellular stress responses [8]. In AAB, sirtuins may also contribute to the regulation of redox balance, protecting cells from damage caused by the accumulation of reactive oxygen species (ROS) during intense metabolic activities. Moreover, sirtuins may be involved in regulating stress responses, especially given the challenging environments these bacteria often encounter, such as fluctuating pH and ethanol concentrations during bioprocess [14]. These enzymes play crucial roles in cellular homeostasis, and their conservation across various domains of life underscores their importance. Further research into these bacterial sirtuins could uncover new pathways that could be harnessed for biotechnological applications, such as improving bioprocess efficiency or enhancing bacterial resistance to industrial stressors.

## 2. Results and Discussion

### 2.1. Sirtuins Are Present in Some Genera of Acetic Acid Bacteria

The results from utilizing the BLAST method from the National Center for Biotechnology Information (NCBI) confirmed the presence of sirtuins in 12 out of 25 genera of AAB (Table 1).

The sirtuins’ sequences of typical bacterial species from the above-mentioned genera include *Acetobacter senegalensis* (Acc. No. WP_058987957.1), *Asaia* sp. VD9 (Acc. No. WP_336760618.1), *Gluconacetobacter liquefaciens* (Acc. No. WP_141288808.1), *Gluconobacter oxydans* (Acc. No. WP_367632865.1), *Kozakia baliensis* (Acc. No. WP_070406037.1), and *Novacetimonas hansenii* (Acc. No. WP_003617640.1) for sirtuins with the SIR2 superfamily domain or shortly SIR2 sirtuins; *Acetobacter aceti* (Acc. No. WP_149026469.1),* Komagataeibacter oboediens* (Acc. No. WP_217319909.1), and *Novacetimonas hanseii* (Acc. No. WP_062809629.1) for sirtuins with the SIR2_2 domain or shortly SIR2_2 sirtuins; and *Acetobacter aceti* (Acc. No. WP_077812739.1),* Ameyamaea chiangmaiensis* (Acc. No. NVN40310.1),* Commensalibacter intestini* (Acc. No. WP_008854500.1),* Endobacter medicaginis* (Acc. No. WP_176626724.1),* Entomobacter blattae* (Acc. No. WP_338030774.1),* Gluconacetobacter liquefaciens* (Acc. No. WP_114726815.1),* Komagataeibacter xylinus* (Acc. No. WP_129551765.1),* Nguyenibacter vanlangensis* (Acc. No. WP_176642093.1), and *Novacetimonas hansenii* (Acc. No. WP_062808700.1) for sirtuins with the PRK00481 domain or shortly PRK00481 sirtuins. The sequences of SIR2 sirtuins, consisting of approximately 351 amino acids, and PRK00481 sirtuins, consisting of about 236 amino acids, are much shorter than the SIR2_2 sirtuins’ sequences, which consist of an average of 1179 amino acids. Most AAB have only one or two types of sirtuins present (Table 2). Interestingly, the genera *Acetobacter* and *Novacetimonas* have sirtuins with each of the three types of domains.

The differences in sirtuin presence across various genera, particularly within the group of AAB, can be attributed to several evolutionary and ecological factors. Genera such as *Acetobacter* and *Novacetimonas* have retained all three identified sirtuin types: SIR2, SIR2_2, and PRK00481. This maintenance may be due to the conservation of ancestral genes that encode these proteins, driven by evolutionary pressures that necessitate their presence for specific metabolic or regulatory functions [15,16]. In contrast, genera like *Gluconacetobacter* and *Komagataeibacter* may have lost certain sirtuin family members, such as SIR2_2, over time. This loss could result from different environmental pressures, mutations, or adaptations that rendered some sirtuins non-essential for survival [17]. The distribution of sirtuins among AAB can also be explained by gene loss or horizontal gene transfer. Some genera may have undergone gene loss, where specific sirtuins were deleted or became non-functional over time. Alternatively, horizontal gene transfer could account for why some genera possess additional sirtuins while others do not [18].

The ecological niches of different AAB genera are related to their sirtuin types’ presence, which reflects their adaptations to specific environments and metabolic needs. *Novacetimonas* is found in various habitats, including soil and plants, and has multiple sirtuin types that may help it thrive in changing conditions [15]. *Acetobacter*, known for vinegar production, lives in sugary and acidic environments like fruits, possibly needing a full set of sirtuins for effective energy metabolism and stress management [16]. *Komagataeibacter* is linked to cellulose production in fruit juices and has probably lost some sirtuin variants, possibly due to its specialization in specific environments [17]. *Gluconacetobacter* also thrives in sugary, acidic areas and may have lost some sirtuins, reflecting its ability to use available resources efficiently [18]. *Asaia* and *Endobacter* are found in flowers and insect guts, showing high specialization, with *Asaia* typically having only one sirtuin type [15,18]. *Ameyamaea*, a genus of red ginger flowers (*Alpinia purpurata*) in Thailand, exhibits robust acetate oxidation and thrives in nutrient-rich environments [19]. *Commensalibacter* is associated with insects, helping digest plant materials and ferment sugars, with sirtuins possibly aiding its energy regulation in the insect gut [17]. *Entomobacter*, also linked to insects, assists in digesting complex carbohydrates, with sirtuins potentially supporting its nutrient processing [15]. *Gluconobacter* thrives in sugary, acidic environments and is known for converting glucose to gluconic acid, with sirtuins being very likely crucial for its metabolic pathways [18]. *Kozakia* shares similar habitats with *Gluconobacter* and thrives in sugar-rich environments, with sirtuins possibly helping it manage energy metabolism [20]. *Nguyenibacter*, though less studied, is believed to inhabit similar environments, with sirtuins possibly indicating its adaptation to acidic and sugary conditions [21].

Incomplete genomic data may also contribute to the observed differences in sirtuin presence among AAB. Some genera may not have fully sequenced or annotated genes, leading to gaps in the identification of sirtuin family proteins. This is particularly true for less-studied genera compared to well-researched ones like *Acetobacter*. Research bias may further exacerbate this issue, as studies often focus on commercially important genera, leaving others underexplored [18].

Moreover, the detection of SIR2_2 variants poses challenges due to limitations in existing bioinformatics tools. Many current tools may need to be optimized for identifying subtle variants, which can hinder the discovery of less-reported sirtuin proteins. For instance, the complexity of genomic regions where these variants reside can lead to inaccuracies in variant calling, particularly in areas with high sequence similarity or repetitive elements [22]. Additionally, the reliance on reference genomes that may not fully represent the genetic diversity of all populations can further complicate the detection of rare variants like SIR2_2 [22]. The presence of low-frequency variants in heterogeneous populations can also obscure detection, as standard algorithms may struggle to differentiate between true variants and sequencing errors. Furthermore, the structural features of SIR2_2, which may differ subtly from other sirtuins, can complicate the alignment and identification processes [23]. As genomic and proteomic technologies advance, the ability to detect such variants is expected to improve, potentially reshaping our understanding of sirtuins across different genera.

In summary, the differences in sirtuin presence among genera like *Acetobacter*, *Gluconacetobacter*, *Komagataeibacter*, and *Novacetimonas* can be attributed to evolutionary divergence, functional specialization, gene loss, horizontal gene transfer, and ecological adaptations. The challenges in detecting specific variants, particularly SIR2_2, highlight the need for improved bioinformatics tools and more comprehensive genomic studies. Though the present work relies solely on in silico analyses rather than wet-lab (in vitro) experiments, the information we compiled from large databases enabled us to develop a straightforward program for detecting SIR2_2 sirtuin variants in AAB. Details regarding this tool and its application can be found in Section 2.4.

On the other hand, according to UniProt [24], only sirtuins with PRK00481 domain can be sorted into Class III. Based on their annotation, it was also determined that the genes responsible for producing NAD^+^-dependent deacylase with conserved PRK00481 domain are located at a locus within the *cobB* gene. This is significant because the bacterium *E. coli* also has sirtuins, which are regulated by the same gene and are classified as Class III sirtuins [8].

**Figure 2:**
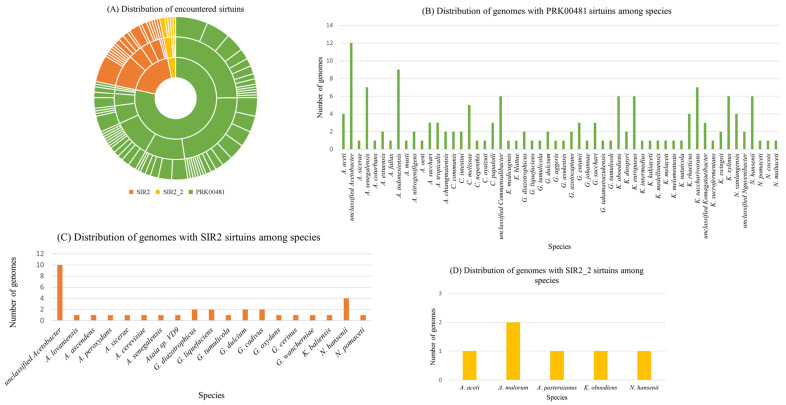
Distribution of encountered sirtuins among AAB species and their genomes. (**A**) The sunburst chart at the top left visually represents the hierarchical distribution of three types of sirtuin protein family models: SIR2 (orange), SIR2_2 (yellow), and PRK00481 (green). The innermost circle categorizes these three types, and as you move outward, each segment represents genera and then species. The size of each segment correlates with the number of genomes containing that particular sirtuin. This chart highlights the diversity and prevalence of these sirtuins across different species. The colors of marked sirtuins in sunburst chart correspond to colors in the clustered column charts. Most species have been found to have PRK00481 sirtuins. (**B**) On the other hand, fewer species have SIR2 and SIR2_2 sirtuins (**C**,**D**). Even the number of genomes is averagely higher when looking at the graph of “Distribution of genomes with PRK00481 sirtuins among species”.

Next, we counted the number of species and genomes with sirtuins from the NCBI BLAST output and calculated the percentage of species and genomes with sirtuins for each type of protein family model. The clustered column charts provide a more detailed breakdown of the distribution of genomes with each type of sirtuin among species of AAB (Figure 2A). The second chart (Figure 2B) stands out because it shows a significantly higher number of genomes with PRK00481 sirtuins among species. The vertical bars are much taller, indicating that PRK00481 sirtuins are more prevalent and widely distributed across the species studied. This suggests that PRK00481 might play a more crucial or universal role in the biological processes of these organisms. The third chart (Figure 2C) shows that fewer species have genomes containing SIR2 sirtuins compared to PRK00481. The vertical bars represent different species, and their height indicates the number of genomes with SIR2 sirtuins. The relatively lower number of genomes suggests that SIR2 sirtuins are less prevalent across the species studied. Like the SIR2 chart, the fourth chart (Figure 2D) shows the distribution of genomes with SIR2_2 sirtuins among species. The pattern is comparable to SIR2, indicating that SIR2_2 sirtuins are also less common across the species. The heights of the bars are generally lower, reinforcing the idea that these sirtuins are not as widespread.

The statistical analysis of sirtuins among AAB reveals several intriguing insights. Descriptive statistics indicate that while a notable percentage of species possess sirtuins, the genomic evidence for these sirtuins is less prevalent (Tables S1–S3). For example, 10.33% of known AAB species possess SIR2 sirtuins, but only 3.44% of their genomes contain these proteins. This discrepancy suggests that even though many species have sirtuins, the detection in their genomes is lower, possibly due to incomplete genomic data or limitations in sequencing technologies. Future research can enhance the completeness of data through more comprehensive genomic sequencing and annotation to ensure more accurate identification and analysis of sirtuins.

Statistical tests, including Fisher’s exact test (Tables S4–S6) and the Kruskal–Wallis test (Tables S7–S9), show that the number and percentage of species and genomes with sirtuins are independent of the genus. This lack of significant difference across genera implies that the presence of sirtuins might be a conserved trait among AAB, reflecting their essential roles in cellular processes such as stress response and metabolic regulation [25]. The uniform distribution of sirtuins across different genera could be attributed to similar ecological adaptations, as these bacteria often occupy comparable niches, such as environments involved in vinegar production and oxidative stress conditions [8].

The correlation analysis (Tables S10–S12) reveals a strong positive relationship between the percentage of species with sirtuins and the percentage of genomes with these sirtuins. This correlation suggests that the evolutionary or functional pressures maintaining sirtuins across species are similarly acting on their genomes. The low variability in the percentage data across genera further supports this, indicating that the proportion of species with sirtuins does not vary significantly enough to produce statistical differences.

Multinomial logistic regression results (Tables S13–S18), with an R^2^McF value of 1.000, indicate a perfect fit, suggesting that the genus significantly influences the presence of sirtuins. This strong predictive power implies that certain genera have evolved specific mechanisms or conditions favoring the presence of these proteins. The significant influence of the genus as a predictor highlights the importance of evolutionary and ecological factors in shaping the distribution of sirtuins among AAB [8].

### 2.2. Structure of Sirtuins in Acetic Acid Bacteria

Protein structures for each sirtuin family model (SIR2, SIR2_2, and PRK00481) of AAB’s typical bacterial species were constructed with AlphaFold 3 [26]. The gathered results are displayed in Table 3.

**Figure A1:**
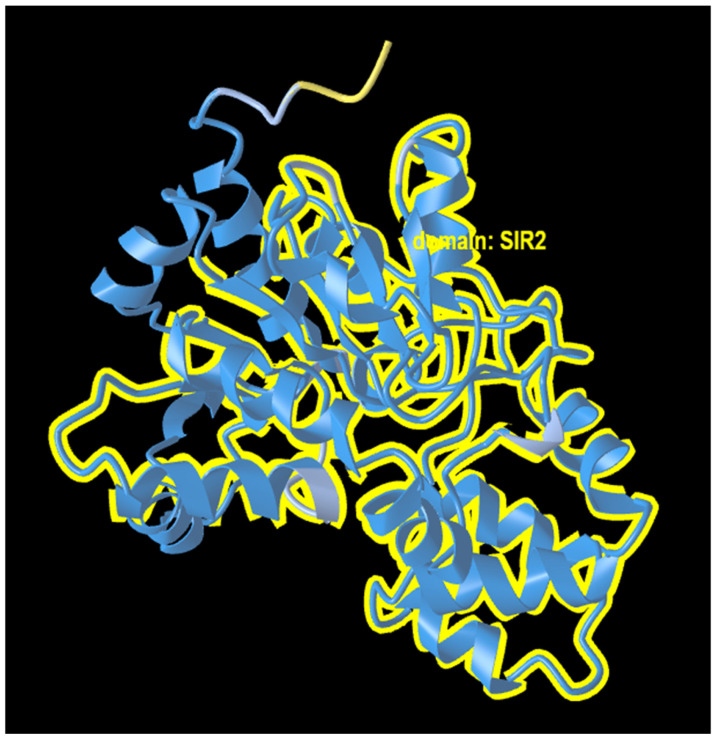
Modelled SIR2 sirtuin (UniProt Acc. No. A0A0U5F059) of *Acetobacter senegalensis*. The yellow border marks SIR2 domain.

**Figure A2:**
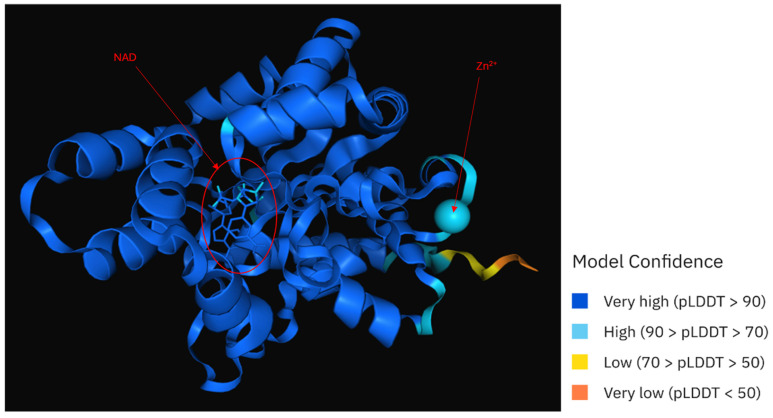
Inserted NAD^+^ and Zn^2+^ in SIR2 sirtuin (UniProt Acc. No. A0A0U5F059) using AlphaFold 3 Beta Server from Google DeepMind.

SIR2 sirtuins are a part of the SIR2 family of proteins and are NAD^+^-dependent deacetylases. This family is highly conserved and has been shown to play roles in stress response, aging, and metabolism [27]. Table 3 shows that all SIR2 sirtuins from various genera bind to NAD^+^ (indicated by “+”), confirming their typical function as NAD^+^-dependent enzymes. This aligns with their known role in deacetylating proteins in an NAD^+^-dependent manner, consistent with SIR2’s primary enzymatic activity. Zinc ion binding is variable across different genera. Some genera like *Acetobacter* and *Gluconacetobacter* bind to zinc (indicated by “+”), while others like *Kozakia* and *Novacetimonas* show partial binding (indicated by “+/−” or “−”). This variability may indicate a functional divergence or modification in the structure that alters Zn^2+^ binding affinity. The structural scores (ipTM and pTM) range from 0.77 to 0.97, indicating high confidence in the predicted protein models for these SIR2 sirtuins. A score closer to 1.0 suggests that the protein’s structure is well-predicted and likely corresponds to the native conformation. *Acetobacter* and *Gluconobacter* have particularly high scores, suggesting their sirtuins are structurally robust and functional. For example, in the modeled SIR2 protein of *Acetobacter senegalensis* (Figure A1 and Figure A2), the NAD^+^ ligand is displayed mostly in dark blue color in a dark blue region, indicating very high confidence in this region’s accuracy. Zn^2+^ has slightly lower confidence, marked with light blue, but still high enough to possibly have an important role in stabilizing the structure. This is because of a histidine located next to zinc, with which an ionic bond is formed. Thus, it results in an SIR2 protein that includes an NAD^+^ binding site but lacks the conserved zinc-binding cysteines [27].

**Figure A3:**
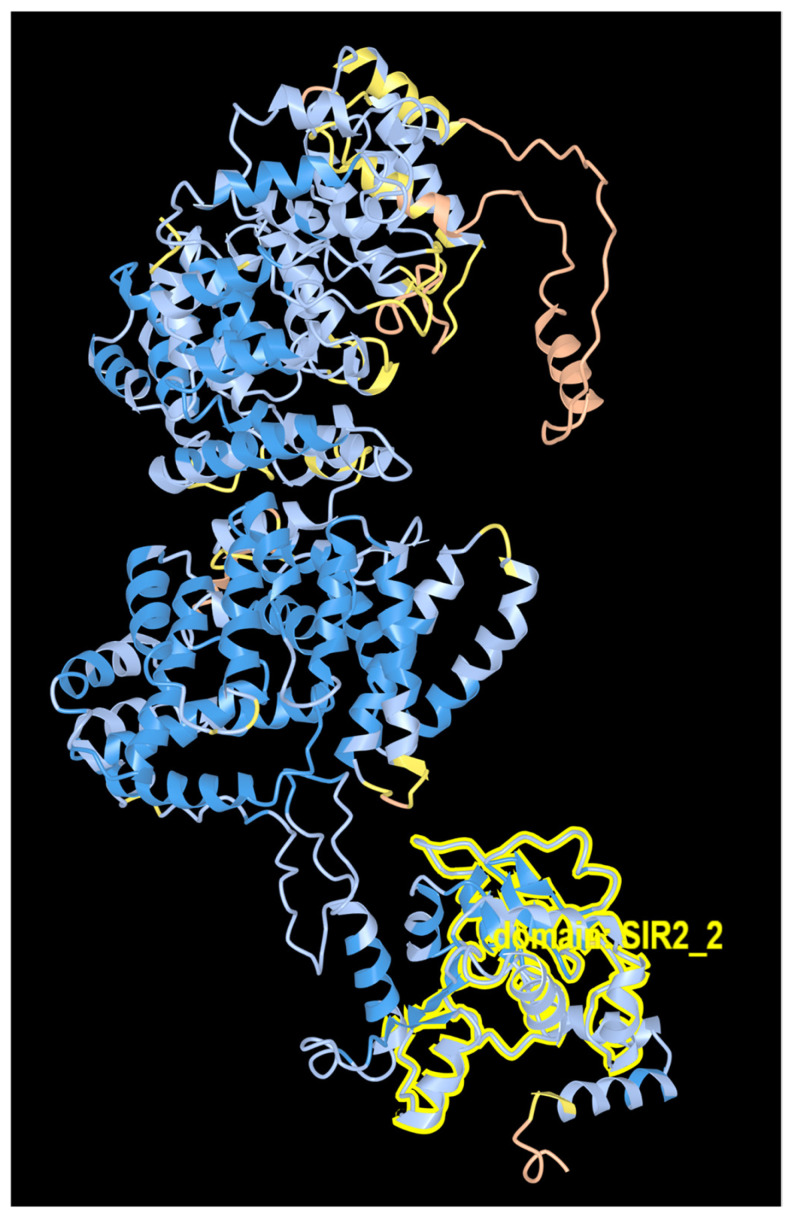
Modelled SIR2_2 sirtuin (UniProt Acc. No. A0A1A0DNT0) of *Acetobacter pasteurianus*. The yellow border marks SIR2_2 domain.

**Figure A4:**
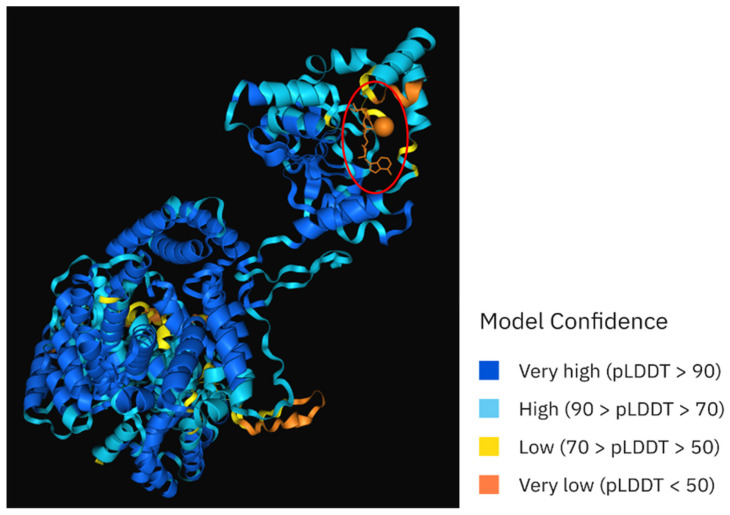
Inserted NAD^+^ and Zn^2+^ in SIR2_2 sirtuin (UniProt Acc. No. A0A1A0DNT0) using AlphaFold 3 Beta Server from Google DeepMind. Both positions of NAD^+^ and Zn^2+^ are marked with a red circled area.

SIR2_2 proteins belong to the SIR2 family but are more similar to the human FAM118B protein. They are also predicted to have NAD^+^-dependent deacylase activity, though their specific roles are less well-defined compared to SIR2 [28]. All SIR2_2 sirtuins in Table 3 also show positive NAD^+^ binding (indicated by “+”), which supports their classification as NAD^+^-dependent enzymes. Their function might resemble the classical deacetylase activity of the SIR2 family. Interestingly, none of the SIR2_2 sirtuins bind to Zn^2+^ ions (all marked “−”). This lack of zinc binding suggests that the SIR2_2 sirtuins either do not require zinc for their catalytic activity or may have evolved alternative mechanisms for stabilization and catalysis, distinct from SIR2 sirtuins. The scores for SIR2_2 range from 0.66 to 0.95. Lower scores in certain genera, such as *Komagataeibacter* (0.66), suggest that the model prediction for these proteins is less confident, potentially indicating structural differences or deviations from canonical sirtuin structures. However, some genera like *Acetobacter* have high scores (0.95), indicating a well-predicted structure similar to SIR2 sirtuins. For instance, in the modeled SIR2_2 protein of *Acetobacter pasteurianus* (Figure A3 and Figure A4), the NAD^+^ ligand and Zn^2+^ are displayed with orange color, meaning they have very low confidence in this region’s accuracy. Thus, they have no function in the SIR2_2 domain and do not bind to the protein. This results in the SIR2_2 protein lacking an NAD^+^ binding site and conserved zinc-binding cysteines. Consequently, it is not an NAD^+^-dependent deacylase [28].

PRK00481 sirtuins are NAD^+^-dependent deacylases and share homology with the *E. coli* CobB protein, a known NAD^+^-dependent protein deacylase. PRK00481 is also part of the SIR2 superfamily and performs functions similar to SIR2 but with potentially broader deacylation activity [29]. All PRK00481 sirtuins in Table 3 show positive NAD^+^ binding (indicated by “+”), reaffirming their dependence on NAD^+^ for deacylation activity. All PRK00481 sirtuins show binding to zinc ions (all marked “+”), suggesting that these sirtuins rely on zinc for their catalytic function. The scores for PRK00481 are consistently high, ranging from 0.87 to 0.93, indicating that these models are well-structured and likely to resemble their functional forms. The consistency in scores suggests a high degree of conservation across genera for this type of sirtuin, especially in species like *Acetobacter* and *Commensalibacter*.

**Figure A5:**
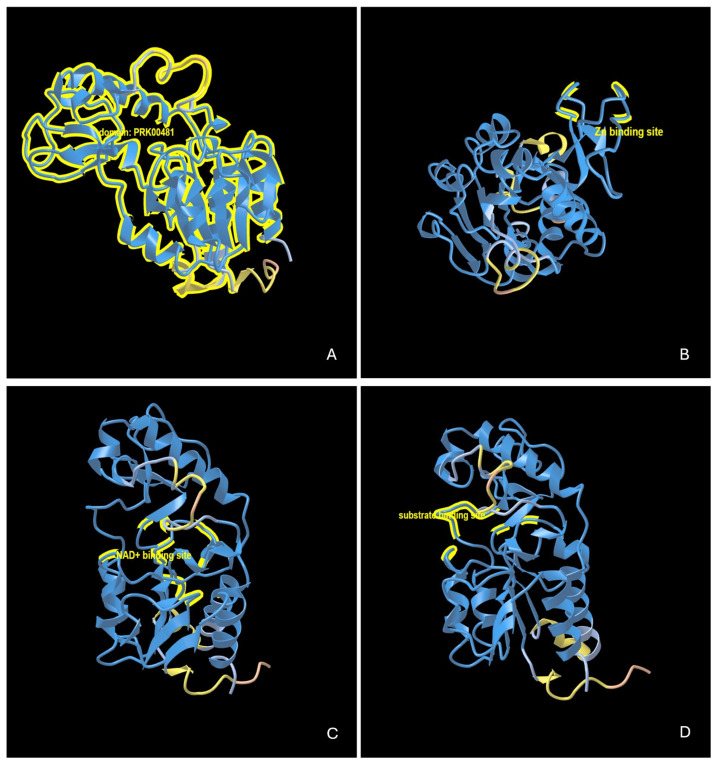
Modelled PRK00481 sirtuin (UniProt Acc. No. A0A252EJJ2) of *Acetobacter senegalensis*. The yellow border marks PRK00481 domain (**A**), Zn^2+^ binding site, which consists of four cysteines (**B**), NAD^+^ binding site (**C**), and substrate binding site (**D**).

PRK00481 domain-containing proteins are NAD^+^-dependent deacylases of the SIR2 family [29], which are better understood than ones with the SIR2 or SIR2_2 domain. These proteins possess a conserved catalytic core domain consisting of approximately 275 amino acid residues. This core is flanked by variable N- and C-terminal regions. The catalytic core maintains a high structural similarity among different sirtuins, characterized by an elongated shape. This structure includes an open α/β Rossmann-fold typical of NAD^+^/NADH-binding proteins and a smaller globular domain formed by two insertions in the Rossmann fold. One insertion binds a structural zinc ion via four conserved cysteine residues, while the other forms a helical module (Figure A5). The enzyme’s active site is situated in a deep cleft between these two regions, accommodating both NAD^+^ and acetyl-lysine substrates. Upon binding the peptide containing the acylated lysine, the substrate’s main chain interacts with two flanking strands, one in the Rossmann fold and the other in a loop with a conserved FGExL motif. This interaction, termed the “β staple”, positions the acetyl-lysine side chain into a hydrophobic tunnel, triggering a conformational change in the enzyme from an open to a closed state. This closed state allows proper NAD^+^ binding, which adopts a “productive conformation” in the hydrophobic C pocket adjacent to the acyl-lysine tunnel [30].

### 2.3. Function and Mechanisms of Sirtuins in Acetic Acid Bacteria

#### 2.3.1. Sirtuins with SIR2 Domain

So far, there is little known about this group of sirtuins in AAB. The UniProt yielded no results when we searched using BLAST. According to NCBI, from which we obtained our reference sequences, SIR2 sirtuins are described as “SIR2 family proteins similar to NAD^+^-dependent deacetylase that catalyze NAD^+^-dependent protein/histone deacetylation, where the acetyl group from the lysine epsilon-amino group is transferred to the ADP-ribose moiety of NAD^+^, producing nicotinamide and the novel metabolite O-acetyl-ADP-ribose” [27].

Furthermore, inspecting the primary protein sequence reveals GxGxxG-like signatures and other conserved residues typical of a Rossmann fold, suggesting strong NAD^+^-binding capacity. The Rossmann fold underlies the catalytic mechanism, positioning NAD^+^ for cleavage during deacylation reactions. These reactions generate nicotinamide, O-acyl-ADP-ribose, and the deacylated substrate, thus regulating the target protein’s structure and function [30].

Additionally, our analysis in AlphaFold 3 showed that *Gluconobacter*, *Kozakia*, and *Novacetimonas* lack a zinc-binding site (Table 3) which is unusual. This may alter their metal sensitivity and give them a more metal-independent mechanism, advantageous for surviving under fluctuating or stressful conditions [7]. Despite this, the possible function of SIR2 sirtuins in AAB remains NAD^+^-dependent deacetylation. With this reaction they can modulate the acetylation states of key metabolic enzymes, transcription factors, or stress-response proteins, thereby fine-tuning pathways for incomplete oxidation of sugars and alcohols. Hence, the protein’s core SIR2 domain strongly suggests a role in regulating metabolism or stress adaptation through reversible lysine deacetylation [7,8].

The regulatory mechanism of SIR2 sirtuins in AAB, as with all sirtuins, relies on NAD^+^. When NAD^+^ levels are high, the protein’s deacylation rate increases, targeting key enzymes for deacylation and thus modulating their function. During stress or heavy metabolic load, cells may experience elevated NADH or depleted NAD^+^, curbing this enzyme’s activity and protecting certain proteins from excessive deacylation. Because these sirtuins are stimulated by NAD^+^, they link deacylation events to the cell’s energetic state. In periods of high respiration (leading to high NAD^+^ regeneration), they could more aggressively remove lysine acyl modifications, potentially optimizing metabolism for robust acetic acid production. Under nutrient deprivation or heavy reductant load (increased NADH), their activity declines, retaining certain acyl modifications that might be protective or help funnel limited resources into stress tolerance pathways [7,8,31,32].

On the other hand, the product of the deacylation reaction, nicotinamide, can bind back to the active site and inhibit further catalysis (product inhibition). This ensures that the enzyme does not indiscriminately deacylate substrates, thus providing a dynamic feedback mechanism tied to NAD^+^ consumption rates [7,8,30].

Furthermore, AAB rely heavily on two-component systems to sense environmental changes (pH, osmolarity) [33]. Phosphorylated response regulators might directly or indirectly alter sirtuin expression or activity. For instance, under severe acid stress, a regulator could enhance sirtuin gene transcription or recruit it to deacylate specific substrates, aligning gene expression with post-translational control.

In summary, SIR2 sirtuins are NAD^+^-dependent deacylase in AAB that likely operate as metabolic and stress-response “orchestrators”. Their deacylation targets critical enzymes, possibly enabling them to optimize incomplete oxidation of sugars/alcohols for acetic acid production, defend against acidic, oxidative, and osmotic insults, and balance the cellular redox environment with dynamic post-translational modification.

#### 2.3.2. Sirtuins with SIR2_2 Domain

The same story for SIR2 sirtuins applies to SIR2_2 sirtuins in AAB. Although they are described as “SIR2 family proteins similar to the human protein FAM118B, which belongs to the SIR2 (silent information regulator-2) superfamily and may be an NAD^+^-dependent deacylase” [28], they exhibit an NAD^+^ binding site but lack Zn^2+^ binding sites (Table 3). The mechanism and precise functions of these sirtuins in AAB remain unclear and warrant further investigation.

However, by inspecting the primary protein sequences and the AlphaFold 3 results, we come to the same conclusions as for the SIR2 sirtuins in AAB, meaning their possible functions, mechanisms, and responses to environmental stress are like the ones described in Section 2.3.1. Though this in silico analysis was based purely on primary protein sequences and AlphaFold 3, we encourage scientists to fill out the holes in the data with in vitro experiments. This will help us all uncover the whole picture of sirtuin functions in AAB.

#### 2.3.3. Sirtuins with PRK00481 Domain

**Figure 3:**
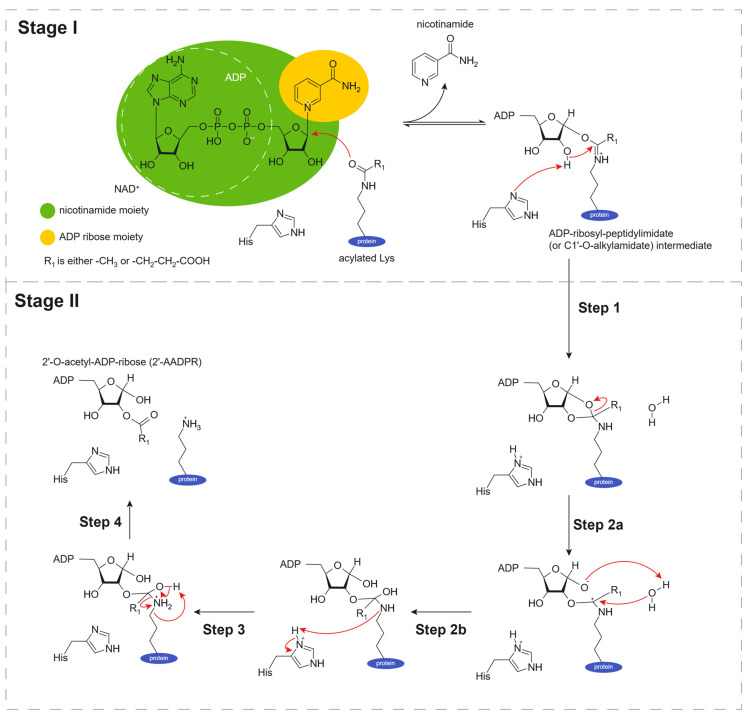
The image depicts the potential catalytic mechanism of NAD^+^-dependent deacetylation by SIR2 family proteins in AAB. This figure has been adapted from Figure 4 in A Molecular Perspective on Sirtuin Activity, *Int. J. Mol. Sci.*, 2020, 21, by Carla S. S. Teixeira et al. [30].

According to automatic annotation provided by UniProt [24], sirtuins with PRK00481 domain in AAB are NAD^+^-dependent lysine deacetylases and desuccinylases that specifically remove acetyl and succinyl groups on target proteins. They modulate the activities of several proteins which are inactive in their acylated form. An important role is played by the cofactor Zn^2+^, where one cation binds to each subunit and stabilizes the enzyme’s structure. Furthermore, two residues (Tyr and Arg) present in a large hydrophobic pocket are probably involved in substrate specificity. They are important for desuccinylation activity, but dispensable for deacetylation activity. The catalytic activity of these enzymes takes its place in the cytoplasm and is represented below by the following reactions [34,35] and their potential mechanism (Figure 3):

H_2_O + N_6_-acetyl-L-lysyl-[protein] + NAD^+^ → 2″-O-acetyl-ADP-D-ribose + L-lysyl-[protein] + nicotinamide

H_2_O + N_6_-succinyl-L-lysyl-[protein] + NAD^+^ → 2″-O-succinyl-ADP-D-ribose + L-lysyl-[protein] + nicotinamide

The potential mechanism of PRK00481 sirtuins in AAB involves two main stages with multiple steps within each stage (Figure 3). In the first stage, activation and formation of the intermediate occur. Initially, NAD^+^ binds to the active site of the SIR2 enzyme. Concurrently, the enzyme binds to the acylated lysine residue on the target protein. The enzyme then catalyzes the cleavage of NAD^+^, releasing the nicotinamide moiety (highlighted in green) and forming an ADP-ribosyl-peptidylimidate (or C1′-O-alkylamidate) intermediate. In the second stage, deacetylation and release of products take place. The intermediate undergoes a rearrangement facilitated by a histidine (His) residue in the enzyme’s active site. Hydrolysis occurs, adding a water molecule to the intermediate, resulting in the formation of a 2′-O-acetyl-ADP-ribose (or 2′-AADPR) intermediate. The acetyl group from the acylated lysine is transferred to the ADP-ribose moiety, forming a stable 2′-AADPR product. Further rearrangement releases the deacetylated lysine residue from the enzyme, and the final product, 2′-AADPR, is released, completing the catalytic cycle [30].

Like other sirtuins, PRK00481 sirtuins in AAB are highly responsive to the cellular NAD^+^ pool. High NAD^+^ availability accelerates the deacylation reaction, whereas low NAD^+^ or high NADH can limit their activity. Besides this nicotinamide (released during deacylation) can inhibit the sirtuin by binding back into the active site, creating a product-based feedback loop [30,31].

Nicotinamide mononucleotide (NMN) has gained significant attention for its role as a precursor to NAD^+^, a vital coenzyme involved in numerous biological processes. NAD^+^ is essential for energy metabolism, DNA repair, and cellular signaling, making it crucial for maintaining cellular health and function. Recent studies have highlighted NMN’s potential to enhance metabolic health and its implications for bacterial metabolism [36]. As research continues to uncover the intricate relationships between NMN, NAD^+^, sirtuins, and bacterial metabolism, it becomes clear that NMN not only influences human health but also plays a significant role in the metabolic activities of various bacteria.

The NAD^+^-dependent protein, PRK00481 sirtuin, deacylase activity pathway of AAB illustrates the steps involved in modifying proteins by removing acyl groups. This process is critical for regulating various cellular functions, including gene expression and metabolic pathways. Enzymes involved in this pathway, such as sirtuins, rely on NAD^+^ to perform their functions effectively. NMN, as a direct precursor to NAD^+^, is integral to this process. When NMN is ingested, it is rapidly converted into NAD^+^ within cells, thereby enhancing the activity of NAD^+^-dependent enzymes. Research indicates that NMN supplementation can suppress aging and enhance metabolic activity, which is particularly relevant in the context of bacterial metabolism. For instance, NMN has been shown to influence gut microbiota, promoting the growth of beneficial bacteria while inhibiting harmful species [37].

The effects of NMN on bacterial metabolism are multifaceted. NMN not only serves as a substrate for NAD^+^ synthesis but also influences the metabolic pathways of bacteria. Studies have shown that NMN can enhance the growth of beneficial gut bacteria such as *Bacteroides*, which are known to produce short-chain fatty acids (SCFAs) like propionate. These metabolites play a crucial role in maintaining gut health and regulating inflammation [37]. Moreover, NMN has been linked to improved bacterial phagocytosis and bactericidal activity in immune cells, enhancing the body’s ability to combat infections [38]. This suggests that NMN may not only support the metabolism of beneficial bacteria but also bolster the host’s immune response, creating a synergistic effect that promotes overall health.

**Figure 4:**
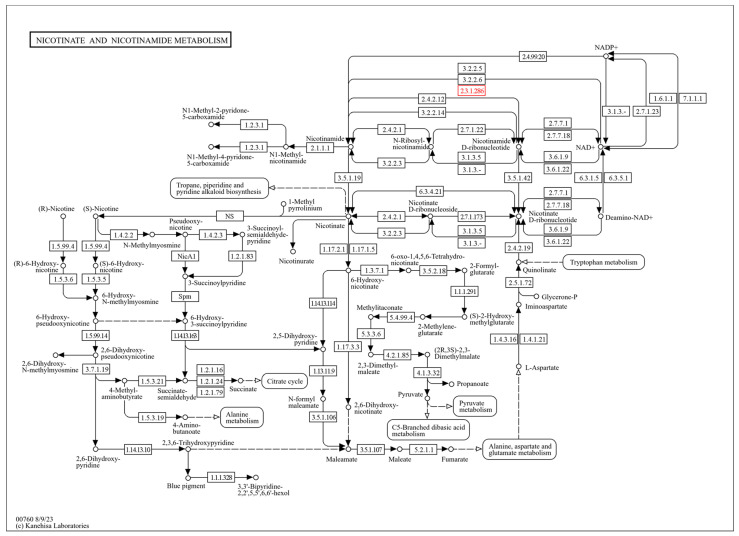
NAD^+^-dependent protein deacylase activity path in nicotinate and nicotinamide metabolism of AAB (specifically *Entomobacter blattae*) identifiable by a red outlined EC 2.3.1.286 [39].

In addition to its effects on gut bacteria, NMN is crucial in the metabolism of nicotinate and nicotinamide in bacteria such as AAB. The KEGG diagram of AAB (specifically *Entomobacter blattae*) detailing these metabolic pathways illustrates the role of NAD^+^-dependent protein deacylase (EC 2.3.1.286), which modifies proteins by removing acyl groups (Figure 4). This enzymatic activity is vital for regulating cellular functions and metabolic pathways within the bacterium. Nicotinate and nicotinamide serve as precursors for NAD^+^, a coenzyme critical for numerous enzymatic reactions, including those involved in energy production and DNA repair. The conversion of nicotinate to NAD^+^ through various enzymatic steps underscores the importance of these metabolites in sustaining cellular functions.

**Figure 5:**
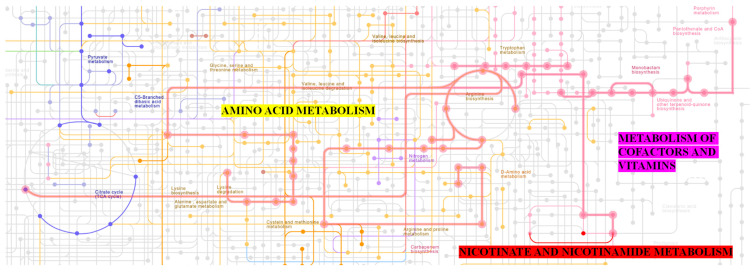
Scheme of metabolic pathways of AAB (specifically *Entomobacter blattae*) [40]. The red line pathway on the right, above the sign “nicotinate and nicotinamide metabolism”, shows NAD^+^-dependent protein deacylase activity, and the red dot above shows nicotinamide mononucleotide (NMN). The nicotinate and nicotinamide metabolism are a part of a larger network of metabolism of cofactors and vitamins. The network metabolism of cofactors and vitamins is displayed with thickened pink lines and dots. This network is deeply entangled with other metabolism processes, especially with the network of amino acid metabolism displayed with yellow lines and dots.

The metabolism of nicotinate and nicotinamide in AAB not only supports energy production but also influences the overall metabolic health of the bacterium (Figure 5). By facilitating the synthesis of NAD^+^, these pathways enable the bacterium to efficiently manage its energy resources and respond to stressors. Furthermore, the activity of NAD^+^-dependent protein deacylase is linked to the regulation of gene expression and protein function, which are crucial for adapting to changing environments, in which AAB also thrive. Research has shown that the modulation of NAD^+^ levels through nicotinate and nicotinamide metabolism can enhance bacterial growth and resilience, particularly in competitive environments where efficient nutrient utilization is vital [36,37]. This means that PRK00481 sirtuins of AAB have a potential role in fighting against environmental stress, such as low pH, oxidative stress, or nutrient deficiency.

In summary, NMN is a vital component in the NAD^+^ biosynthesis pathway, significantly impacting both human health and the metabolism of AAB. By enhancing NAD^+^ levels, NMN supports the activity of PRK00481 sirtuins. Furthermore, NMN’s ability to influence the metabolic pathways of AAB underscores its potential as a therapeutic agent for improving metabolic health and combating environmental stress. As research continues to explore the connections between NMN, NAD^+^, sirtuins, and bacterial metabolism, it is clear that NMN and PRK00481 sirtuins hold promise for enhancing the health and functionality of these important microorganisms.

### 2.4. Classification of Acetic Acid Bacteria’s Sirtuins

**Figure 6:**
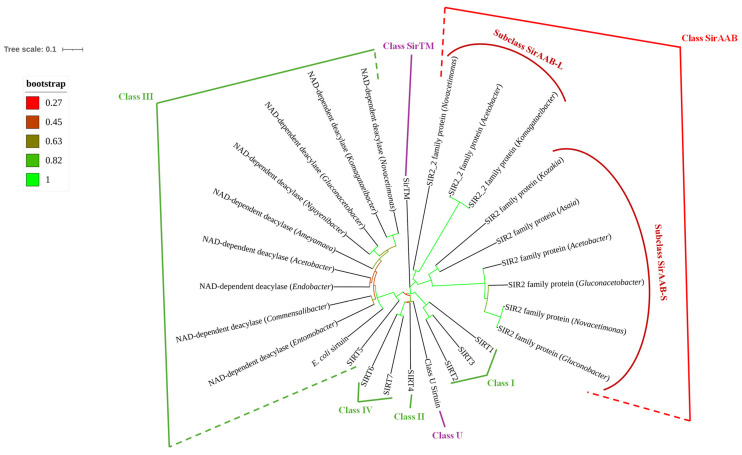
Unrooted phylogenetic tree representing positions of AAB sirtuins among representatives of established classes of sirtuins. Sirtuins labeled from SIRT1 to SIRT7, Class U sirtuin, *E. coli* sirtuin, and SirTM represent the reference sequences, which are surrounded by sequences of different AAB genera. SIRT1-SIRT7 represent human sirtuins, Class U sirtuin is represented by *Geobacillus* sp., and the SirTM sirtuin by *Streptococcus* sp. PRK00481 sirtuins of AAB are labeled as “NAD^+^-dependent deacylases” and classified together with *E. coli* sirtuin and SIRT5 into Class III of sirtuins. SIR2_2 family proteins and SIR2 family proteins are not classified into the known classes of sirtuins, thus we labeled them together as Class SirAAB and separately as subclasses SirAAB-L and SirAAB-S. The accession numbers of sirtuins used for the phylogenetic construction are listed in Section 3.5. The evolutionary history was inferred using the Neighbor-Joining method [6]. The optimal tree is shown. The tree is drawn next to the tree scale (upper left corner), with branch lengths in the same units as those of the evolutionary distances used to infer the phylogenetic tree. The scale length is 0.1, which corresponds to 10 substitutions per 100 positions or 10% sequence divergence in the phylogenetic tree. The bootstrap values (ranging from 0 to 1) are presented by colored branches, with green branches having the highest bootstrap values and red branches having the lowest bootstrap values. A legend for bootstrap values is provided under the tree scale. The evolutionary distances were computed using the p-distance method and are in the units of the number of amino acid differences per sequence. The rate variation among sites was modeled with a gamma distribution (shape parameter = 1). This analysis involved 28 amino acid sequences. All ambiguous positions were removed for each sequence pair (pairwise deletion option). There were a total of 386 positions in the final dataset.

A phylogenetic analysis of sirtuins in AAB highlights the evolutionary diversity of these proteins. Figure 6 illustrates a circular phylogenetic tree of sirtuins, depicting relationships among sirtuins of AAB genera and representatives of different classes. Bootstrap values (indicating the robustness of each clade) provide confidence in these evolutionary relationships.

Sirtuins are classified into several known classes based on their sequence homology and functional characteristics [3,32]. Class I includes SIRT1, SIRT2, and SIRT3. These sirtuins are involved in deacetylation processes and play roles in metabolic regulation, DNA repair, and cellular stress response [25]. Class II is primarily represented by SIRT4, which is known for its role in regulating mitochondrial functions. Although SIRT4’s deacetylase activity is less pronounced, it plays a critical role in controlling metabolic homeostasis by regulating glutamate dehydrogenase [41]. Class III group contains NAD^+^-dependent deacetylases of AAB and includes SIRT5, along with sirtuin of *E. coli* CobB. Their high bootstrap support on the phylogenetic tree indicates a well-supported evolutionary lineage, suggesting that these proteins are highly conserved across different species, including AAB. SIRT5 is known for its role in desuccinylation and demalonylation [7], which are critical for cellular metabolic processes, particularly in AAB that frequently encounter metabolic stress during bioprocess [14]. Class IV includes SIRT6 and SIRT7, which have been shown to participate in DNA repair, regulation of metabolic homeostasis, and stress responses. SIRT6 is involved in maintaining genome stability, a crucial feature for bacterial survival under stressful conditions [7]. Class U sirtuins, while less characterized, have been identified across various bacterial species. They represent a divergent functional group with specialized roles in bacterial physiology, possibly related to environmental adaptation [5]. Class SirTM includes the SirTM proteins, which are distinct due to their involvement in telomere maintenance. Although primarily studied in eukaryotes, related proteins in bacteria could serve roles in genomic integrity and protection under stress [5].

The right side of the phylogenetic tree represents the as-yet undescribed class of sirtuins (Figure 6). This class represents a novel evolutionary lineage. It diverges significantly from other classes based on its phylogenetic placement, suggesting it may have unique structural or functional characteristics. Notably, this new class lacks a macrodomain, which differentiates it from Class SirTM, known for its association with genomic stability. Within this new group, two strongly supported clades exist. The first clade consists of SIR2 proteins from species of the genera *Acetobacter*, *Asaia*, *Gluconacetobacter*, *Gluconobacter*, *Kozakia*, and *Novacetimonas*. The second clade comprises SIR2_2 proteins from *Acetobacter*, *Komagataeibacter*, and *Novacetimonas*. SIR2_2 sirtuins, while structurally like SIR2, may have evolved distinct functions, potentially adapting to the specific stressors encountered by these bacteria. These two distinctive evolutionary lineages could represent two subclasses of sirtuins, each with specific roles in stress adaptation and regulation of metabolic processes in AAB.

**Figure 7:**
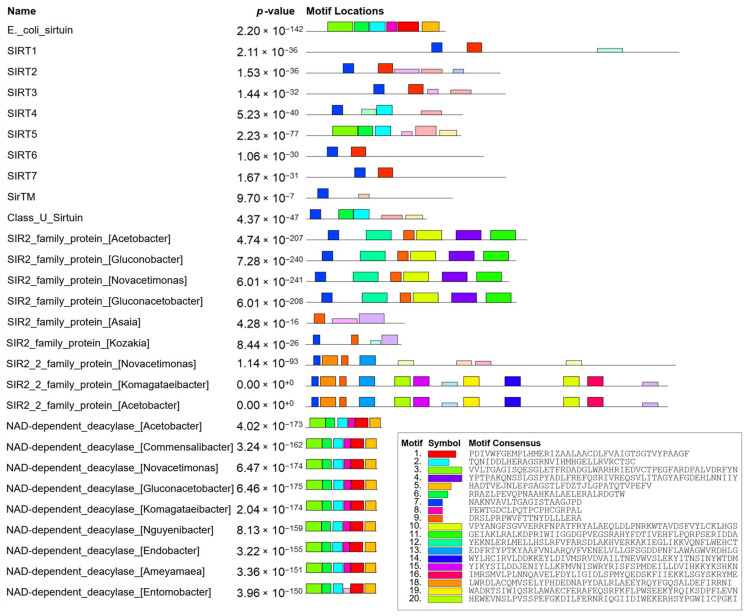
Results from MEME tool with 20 identified motifs in representative sirtuin sequences and their respective *p*-values.

Moreover, the MEME tool [42] results provide us with a detailed analysis of the motifs found in the sirtuins from various organisms. The sirtuins used for the construction of the phylogenetic tree are listed in the left column (Figure 7). In the middle column are the *p*-values. *p*-value indicates the statistical significance of the motifs identified in each protein. Lower *p*-values suggest higher confidence that the motif is not found by chance. Extremely low *p*-values (e.g., 2.20 × 10^−142^ for E._coli_sirtuin) indicate highly significant motifs. All *p*-values listed are very low, suggesting that the motifs identified are highly significant across all proteins. On the right side are the motif locations. This section shows the locations of the identified motifs within each protein sequence. Each colored box represents a different motif. The alignment and pattern of these motifs can indicate conserved regions. The legend at the bottom right shows the motif symbols and their corresponding consensus sequences. These consensus sequences represent the most common sequence pattern for each motif across the proteins analyzed (Figure 7).

**Figure 8:**
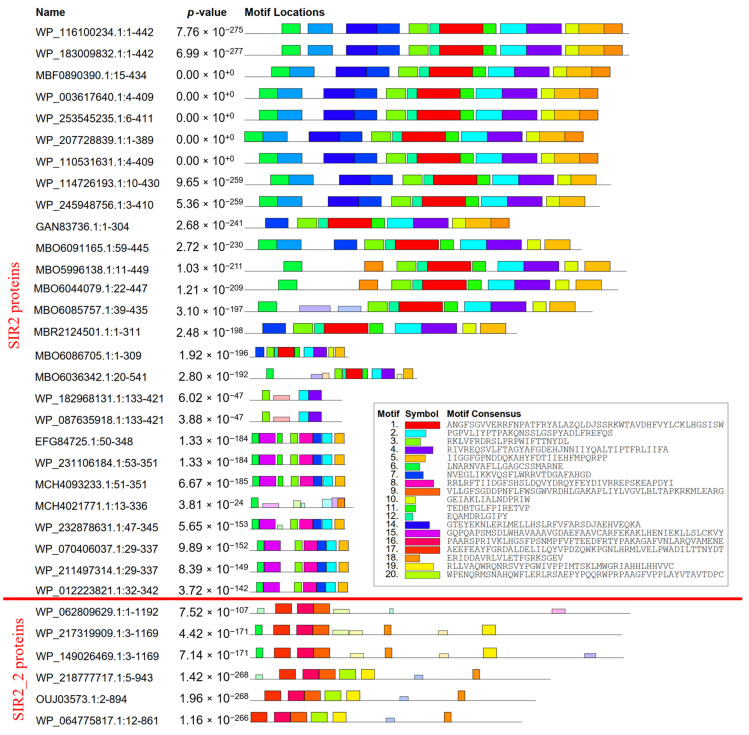
Comparison between SIR2_2 and SIR2 protein motifs. The red line separates SIR2 proteins (above the line) from SIR2_2 proteins (below the line).

Comparing sequences of the new class (from SIR2_family_protein_[Acetobacter] to SIR2_2_family_protein_[Acetobacter]), we notice one motif occurring just in those nine sequences (Figure 7). It is the ninth motif (Figure S1), with an E-value of 3.9 × 10^−57^ and a width of 21. Therefore, it provides valuable information that this class of sirtuins is distinctive from others, making it a class of its own. Besides the previous comparison, the SIR2_2_family_protein group was compared to the SIR2_family_protein group. In SIR2_2 proteins, only the 13th motif is present and absent in all other sequences (Figure S2). This shows that there is a division inside the new class into two new subclasses. If we also consider the bootstrap values of those two separate lineages, it can be stated that there are two subclasses in the new class. Thus, this led us to compare the two subclasses with a larger sample (Figure 8). We can clearly see that they differ in three larger motifs—motifs 9, 16, and 17 (Figures S3–S5), which are only present in SIR2_2 proteins but not in SIR2 proteins, and one smaller motif—motif 3 (Figure S6), which is only present in SIR2 proteins but not in SIR2_2 proteins. Although the SIR2 protein subclass is more diverse in having more motifs than SIR2_2 proteins, they are similar in the second motif which is absent in SIR2_2 proteins (Figure 8).

Based on the phylogenetic analysis and the clear distinction in amino acid sequence, we propose naming the new class “SirAAB”, reflecting their identity as sirtuins derived from AAB. Furthermore, to accommodate the observed subgroup differences, we suggest dividing SirAAB into two subclasses: “SirAAB–S” (representing the shorter sequence variants) and “SirAAB–L” (representing the longer sequence variants). This nomenclature preserves the established SIR2 naming convention while underscoring the unique characteristics of these newly identified sirtuin subclasses.

Moreover, addressing the problem with SIR2_2 variants in Section 2.1, we have designed a simple program/tool based on the motif discovery, which can be found in the Supplementary Materials. This Python program identifies whether a given protein sequence is likely to be an SIR2_2 sirtuin variant of the AAB by checking for the presence of three known motifs at a high level of similarity (default threshold: 90%).

Firstly, the program defines a dictionary called “motifs” with unique integer keys and corresponding motif sequences as values. In it, there are three motifs, with IDs 9 (Figure S3), 16 (Figure S4), and 17 (Figure S5) (that corresponds to the article’s motifs in Figure 8), each representing a specific region that is important for classifying the query protein as an SIR2_2 sirtuin variant of the AAB.

Secondly, the code uses “PairwiseAligner” from the Biopython library to perform a global alignment between the query sequence and each motif sequence. It then computes a “motif identity score” (alignment score divided by the length of the motif) to see if the query sequence meets or exceeds the similarity threshold (default: 0.9).

Finally, if all motif IDs in the dictionary are matched (meaning the query sequence is at least 90% similar to each defined motif), the program lists all the matched motifs in squared brackets and concludes that the sequence is an SIR2_2 sirtuin variant of AAB. Otherwise, it reports that the sequence does not match all required motifs. It lists the matched ones in the square brackets, or it can leave the brackets empty, meaning no motifs were matched. Thus, it concludes that this is not an SIR2_2 sirtuin variant of the AAB.

## 3. Materials and Methods

### 3.1. Analysis Using NCBI BLAST

Starting in the NCBI database, we searched for protein family models [43] of sirtuins by searching for the word SIR2 and choosing the filter protein family models. Out of 35 hits, three protein family models were found to have at least one of the AAB on their list of “Sequences with this architecture”: SIR2 family protein with SIR2_2 conserved domain (accession ID: pfam13289; further mentioned as SIR2_2 protein), NAD^+^-dependent deacylase with PRK00481 conserved domain (accession ID: PRK00481; further mentioned as PRK00481 protein) and SIR2 family protein with SIR2 conserved domain (accession ID: cl00195; further mentioned as SIR2 protein) [27,28,29]. Each of these protein family models has a representative amino acid sequence in AAB. Fourteen sequences were obtained (see below) and used as input for systematic searching of sirtuins among all available genomes of AAB using the blastp algorithm with default settings [44,45]. WP_062809629.1 was used to find sirtuins with the SIR2_2 domain, WP_116100234.1 and CEF41304.1 were used to find sirtuins with the SIR2 domain, and lastly WP_086897390.1, KXV56507.1, KXV49631.1, WP_110439258.1, PXZ00335.1, CAI3942800.1, PYD83574.1, WP_010514307.1, SAY47833.1, WP_075595036.1, and CUW46662.1 were used to find sirtuins with the PRK00481 domain. The genomes of the following genera of AAB were analyzed: *Acetobacter* (taxid:434), *Acidomonas* (taxid:34000), *Ameyamaea* (taxid:442968), *Aristophania* (taxid:2697033), *Asaia* (taxid:91914), *Bombella* (taxid:1654741), *Brytella* (taxid:2959299), *Commensalibacter* (taxid:1079922), *Endobacter* (taxid:1649268), *Entomobacter* (taxid:2762277), *Gluconacetobacter* (taxid:89583), *Gluconobacter* (taxid:441), *Granulibacter* (taxid:364409), *Komagataeibacter* (taxid:1434011), *Kozakia* (taxid:153497), *Neoasaia* (taxid:320496), *Neokomagataea* (taxid:1223423), *Nguyenibacter* (taxid:1519186), *Novacetimonas* (taxid:2919364), *Oecophyllibacter* (taxid:2558360), *Saccharibacter* (taxid:231052), *Sorlinia* (taxid:3081148), *Swaminathania* (taxid:209260), *Swingsia* (taxid:1649499), and *Tanticharoenia* (taxid:444052).

The results were filtered based on Percent Identity, E-value, and Query Coverage with the next respective values: Percent Identity 25–100%, E-value: 0–1 × 10^−9^, Query Coverage: 60–100%. The lower threshold of Percent Identity (25%) allows for the inclusion of more distantly related sequences, which might still share functional or structural similarities, and the upper threshold (100%) ensures that identical sequences are included, which is crucial for identifying exact matches. E-value indicates the number of hits one can expect to see by chance when searching a database of a particular size. The range 0–1 × 10^−9^ includes only highly significant matches. An E-value of 1 × 10^−9^ means there is a one in a billion chance that the match is due to random chance, ensuring that the results are highly reliable. The lower threshold of Query Coverage (60%) ensures that a substantial portion of the query sequence is aligned, which is important for meaningful comparisons, and the upper threshold (100%) ensures that the entire query sequence is considered, capturing complete alignments.

### 3.2. Statistical Analysis with Jamovi

Species of AAB with a detected sirtuin were counted according to the official species list in the List of Prokaryotic names with Standing in Nomenclature (LPSN) [46]. The data for the total number of species of each genus were acquired from LPSN and the total number of genomes of each genus was acquired from the NCBI Genome dataset. The percentage of species with sirtuins was calculated as a quotient between the number of species with sirtuins and the number of all species, whereas the percentage of genomes with sirtuins was calculated as a quotient between the number of genomes with sirtuins and the number of all genomes. Afterward, the program Jamovi [47,48,49] was used for statistical analysis of data retrieved from BLAST results. For each of the three sirtuin family models, four statistical tests were performed: Fisher’s exact test, the Kruskal–Wallis test, correlation analysis, and multinomial logistic regression. The confidence interval of all tests was 95%.

Fisher’s exact test was used to examine if there is a significant relationship between different genera and the presence of sirtuins across species or genomes because we have a small number of detections. Two hypotheses were set: the null hypothesis stated that “The number of species and genomes with sirtuins is independent of the genus” and the alternative hypothesis stated that “The number of species and genomes with sirtuins is associated with the genus”.

The Kruskal–Wallis test was used to compare the distribution of the number and percentage of species with sirtuins and the distribution of the number and percentage of genomes with sirtuins across genera. Two hypotheses were set: the null hypothesis stated that “There is no difference in the distribution of number and percentage of species and the number of genomes with sirtuins across different genera” and the alternative hypothesis stated that “There is a significant difference in the distribution of number and percentage of species and the number of genomes with sirtuins across different genera”.

Correlation analysis was used to examine the relationship between the percentage of species with sirtuins and the percentage of genomes with sirtuins. Two hypotheses were set: the null hypothesis stated that “There is no correlation between the percentages of species and percentages of genomes with sirtuins”, and the alternative hypothesis stated that “There is a correlation between the percentages of species and percentages of genomes with sirtuins”.

Multinomial logistic regression was used to predict the presence of sirtuins based on the genus. Two hypotheses were set: the null hypothesis stated that “The predictor (genus) does not influence the presence of sirtuins”, and the alternative hypothesis stated that “The predictor (genus) significantly influences the presence of sirtuins”.

### 3.3. Protein Modeling with AlphaFold

Amino acid sequences of each type of sirtuin, from each species, with the lowest E-value and the highest Query Coverage and Percent Identity were chosen to have their 3D structure constructed by AlphaFold 3 Beta Server from Google DeepMind [26,50]. If there was an AlphaFold 3D Structure link of a 3D protein structure provided in BLAST results, in the subsection of alignments, we also used it to extract the information about domains [51].

The input in AlphaFold Server consisted of three entities: one copy of the NAD^+^ ligand, one copy of the Zn^2+^ ion, and one copy of the amino acid sequence. The inserted amino acid sequences of AAB were WP_077812739.1, NVN40310.1, WP_008854500.1, WP_176626724.1, WP_338030774.1, WP_114726815.1, WP_129551765.1, WP_176642093.1, WP_062808700.1., CEF41304.1, WP_336760618.1, WP_141288808.1, WP_245948756.1, WP_367632865.1, WP_070406037.1, WP_003617640.1, WP_149026469.1, WP_217319909.1, WP_062809629.1, OUJ03573.1, and WP_064775817.1.

### 3.4. Analysis Using UniProt BLAST

WP_086897390.1, WP_116100234.1, and WP_062809629.1 amino acid sequences underwent UniProt BLAST tool with blastp algorithm to provide us with more valuable information about these proteins [24]. The parameters were set to default. Only the search for NAD^+^-dependent deacylase with PRK00481 conserved domain was successfully processed and outputted.

### 3.5. Sequence Alignment and Phylogenetic Analysis Using ClustalW and MEGA11

Sirtuins’ amino acid sequences for each genus of AAB with the overall lowest E-value (WP_116100234.1, MBF0890390.1, WP_003617640.1, WP_114726193.1, WP_188427487.1, WP_070406037.1, WP_062809629.1, WP_217319909.1, WP_149026469.1, WP_061472745.1, WP_034339921.1, WP_113594927.1, WP_183009761.1, WP_078526748.1, WP_342628943.1, WP_176626724.1, WP_212647850.1, and WP_338030774.1), along with the representatives of sirtuin classes (STJ55569.1, NP_036370.2, NP_036369.2, NP_036371.1, NP_001372662.1, NP_001363727.1, NP_057623.2, XP_008011482.1, WP_002992884.1, and WP_011230933.1), were input into the MEGA11 program [52] and used to construct the phylogenetic tree shown in Figure 6. The sequences underwent alignment by the ClustalW algorithm with default settings and were also manually trimmed. Afterward, we used the Models function to find a suitable substitution model for the Neighbor-Joining Tree method [6,53]. The same procedure and method were used to construct the phylogenetic tree in Figure 1 [54], in which the accession numbers are also displayed. After this, both trees were edited in iTol for better visualization [55].

### 3.6. Identifying Sequence Motifs with MEME Suite

The MEME tool from MEME Suite 5.5.5 version [42] was used to perform motif discovery on protein sequences of sirtuins from AAB, *Homo sapiens*, *E. coli*, Class U sirtuin representative, and Class SirTM representative. The submission form was left on default, apart from the number of motifs being set to 20. Accession numbers for the results shown in Figure 7 are available in Section 3.5, while in Figure 8 the accession numbers of aligned protein sequences are already displayed.

### 3.7. Sequence Alignment and Similarity Scoring in Python

A custom Python script was developed using the Biopython library [56] to perform global pairwise alignments between the query protein sequence and each of the motifs. Specifically, Biopython’s “PairwiseAligner” class was utilized in its “global” approach. This approach ensures that the alignment spans the entire length of both the query and motif sequences, providing a comprehensive assessment of similarity.

Upon completion of each global alignment, an alignment score is computed. To standardize scores across motifs of different lengths, this raw alignment score is divided by the length of the motif, yielding a motif identity score. A threshold (default value set at 0.90) is then applied to determine whether the query sequence exhibits sufficiently high similarity to the motif. If the motif identity score meets or exceeds the threshold, the query sequence is deemed a match for that particular motif.

## 4. Conclusions

The in silico analysis provides compelling evidence that sirtuins in AAB are more than mere metabolic regulators. They appear to help these bacteria to cope with stress, particularly under acidic conditions, and may play critical roles in energy management. Based on these findings, we propose a novel sirtuin class specific to AAB, Class SirAAB, which is further subdivided into two subclasses: Subclass SirAAB-L and Subclass SirAAB-S, reflecting their distinct sequence features. To support the study of this specific group of SIR2_2 type sirtuins, a new program was developed, enabling researchers to more easily detect and analyze SIR2_2 variants in AAB.

Overall, this research highlights the potential role of sirtuins in AAB survival and paves the way for optimizing industrial bioprocesses and advancing microbial biotechnology.

## Figures and Tables

**Table 1:** Occurrence of sirtuins among genera of acetic acid bacteria.

Genus	Sirtuins Present (+)/Absent (−)
*Acetobacter*	+
*Acidomonas*	−
*Ameyamaea*	+
*Aristophania*	−
*Asaia*	+
*Bombella*	−
*Brytella*	−
*Commensalibacter*	+
*Endobacter*	+
*Entomobacter*	+
*Gluconacetobacter*	+
*Gluconobacter*	+
*Granulibacter*	−
*Komagataeibacter*	+
*Kozakia*	+
*Neoasaia*	−
*Neokomagataea*	−
*Nguyenibacter*	+
*Novacetimonas*	+
*Oecophyllibacter*	−
*Saccharibacter*	−
*Sorlinia*	−
*Swaminathania*	−
*Swingsia*	−
*Tanticharoenia*	−

**Table 2:** Occurrence of sirtuin type in genera of acetic acid bacteria.

Genus	Type of Protein Family Model		
	SIR2	SIR2_2	PRK00481
*Acetobacter*	+	+	+
*Ameyamaea*			+
*Asaia*	+		
*Commensalibacter*			+
*Endobacter*			+
*Entomobacter*			+
*Gluconacetobacter*	+		+
*Gluconobacter*	+		
*Komagataeibacter*		+	+
*Kozakia*	+		
*Nguyenibacter*			+
*Novacetimonas*	+	+	+

**Table 3:** Results summary of sirtuins’ structural predictions from AlphaFold 3 Beta Server. The table provides information about different types of sirtuins (SIR2, SIR2_2, and PRK00481) from various bacterial genera, with data on their predicted binding affinities to NAD^+^ and zinc ions (Zn^2+^ ion), as well as structural confidence scores (ipTM and pTM).

Sirtuin Domain	Genus	ipTM	pTM	NAD^+^	Zn^2+^ ion
SIR2	*Acetobacter*	0.96	0.93	+	+
	*Asaia*	0.96	0.94	+	+
	*Gluconacetobacter*	0.95	0.93	+	+/−
	*Gluconobacter*	0.97	0.86	+	−
	*Kozakia*	0.85	0.77	+/−	−
	*Novacetimonas*	0.96	0.86	+	−
SIR2_2	*Acetobacter*	0.94	0.65	+/−	−
	*Komagataeibacter*	0.95	0.66	+	−
	*Novacetimonas*	0.93	0.75	+	−
PRK00481	*Acetobacter*	0.92	0.92	+	+
	*Ameyamaea*	0.92	0.93	+	+
	*Commensalibacter*	0.90	0.91	+	+
	*Endobacter*	0.88	0.91	+	+
	*Entomobacter*	0.89	0.90	+	+
	*Gluconacetobacter*	0.93	0.92	+	+
	*Komagataeibacter*	0.87	0.91	+	+
	*Nguyenibacter*	0.81	0.80	+	+
	*Novacetimonas*	0.89	0.90	+	+

## Data Availability

Data are contained within the article.

## Supplementary Materials

The following supporting information can be downloaded at: https://www.mdpi.com/article/10.3390/molecules30030635/s1, Table S1: Descriptive statistics of SIR2 sirtuins; Table S2: Descriptive statistics of SIR2_2 sirtuins; Table S3: Descriptive statistics of PRK00481 sirtuins; Table S4: Results of Fisher’s exact test for number of species and number of genomes with SIR2 sirtuins; Table S5: Results of Fisher’s exact test for number of species and number of genomes with SIR2_2 sirtuins; Table S6: Results of Fisher’s exact test for number of species and number of genomes with PRK00481 sirtuins; Table S7: Results of Kruskal–Wallis test for number of species and number of genomes with SIR2 sirtuins and for percentage of species and percentage of genomes with SIR2 sirtuins; Table S8: Results of Kruskal–Wallis test for number of species and number of genomes with SIR2_2 sirtuins and for percentage of species and percentage of genomes with SIR2_2 sirtuins; Table S9: Results of Kruskal–Wallis test for number of species and number of genomes with PRK00481 sirtuins and for percentage of species and percentage of genomes with PRK00481 sirtuins; Table S10: Results of correlation analysis between percentage of species with SIR2 sirtuins and percentage of genomes with SIR2 sirtuins; Table S11: Results of correlation analysis between percentage of species with SIR2_2 sirtuins and percentage of genomes with SIR2_2 sirtuins; Table S12: Results of correlation analysis between percentage of species with PRK00481 sirtuins and percentage of genomes with PRK00481 sirtuins; Table S13: Results of multinomial logistic regression for number of species with SIR2 sirtuins; Table S14: Results of multinomial logistic regression for number of genomes with SIR2 sirtuins; Table S15: Results of multinomial logistic regression for number of species with SIR2_2 sirtuins; Table S16: Results of multinomial logistic regression for number of genomes with SIR2_2 sirtuins; Table S17: Results of multinomial logistic regression for number of species with PRK00481 sirtuins; Table S18: Results of multinomial logistic regression for number of genomes with PRK00481 sirtuins; Figure S1: Ninth motif of Figure 7; Figure S2: Thirteenth motif of Figure 7; Figure S3: Ninth motif of Figure 8; Figure S4: Sixteenth motif of Figure 8; Figure S5: Seventeenth motif of Figure 8; Figure S6: Third motif of Figure 8; SIR2_2 Sirtuin variant detector of the acetic acid bacteria.
