# Supplementary material for: In Silico Characterization of Sirtuins in Acetic Acid Bacteria Reveals a Novel Phylogenetically Distinctive Group
Source: Molecules. 2025 Jan 31;30(3):635. doi: 10.3390/molecules30030635 (PMC11820453; doi:10.3390/molecules30030635)
Supplement: Supplementary file 1 [file molecules-30-00635-s001.zip › molecules-3390391-supplementary_proofread.pdf]

Table S1. Descriptive statistics of SIR2 sirtuins.

Genus	Number of species with SIR2 sirtuins	Number of genomes with SIR2 sirtuins	Number of all species (child taxa)	Number of all genomes	Percentage of species with SIR2 sirtuins (%)	Percentage of genomes with SIR2 sirtuins (%)
Acetobacter	7	14	58	366	12.07	3.83
Acidomonas	0	0	1	6	0.00	0.00
Ameyamaea	0	0	1	3	0.00	0.00
Aristophania	0	0	1	2	0.00	0.00
Asaia	1	1	10	68	10.00	1.47
Bombella	0	0	8	23	0.00	0.00
Brytella	0	0	1	2	0.00	0.00
Commensalibacter	0	0	9	62	0.00	0.00
Endobacter	0	0	1	3	0.00	0.00
Entomobacter	0	0	1	1	0.00	0.00
Gluconacetobacter	4	5	28	36	14.29	13.89
Gluconobacter	4	4	28	148	14.29	2.70
Granulibacter	0	0	1	10	0.00	0.00
Komagataeibacter	0	0	19	83	0.00	0.00
Kozakia	1	1	1	6	100.00	16.67
Neoasaia	0	0	1	3	0.00	0.00
Neokomagataea	0	0	3	6	0.00	0.00
Nguyenibacter	0	0	1	3	0.00	0.00
Novacetimonas	2	5	4	25	50.00	20.00
Oecophyllibacter	0	0	1	3	0.00	0.00
Saccharibacter	0	0	1	8	0.00	0.00
Sorlinia	0	0	1	1	0.00	0.00
Swaminathania	0	0	1	2	0.00	0.00
Swingsia	0	0	1	1	0.00	0.00
Tanticharoenia	0	0	2	2	0.00	0.00
All	19	30	184	873	10.33	3.44

Table S2. Descriptive statistics of SIR2_2 sirtuins.

Genus	Number of species with SIR2_2 sirtuins	Number of genomes with SIR2_2 sirtuins	Number of all species (child taxa)	Number of all genomes	Percentage of species with SIR2_2 sirtuins (%)	Percentage of genomes with SIR2_2 sirtuins (%)
Acetobacter	3	4	58	366	5.17	1.09
Acidomonas	0	0	1	6	0.00	0.00
Ameyamaea	0	0	1	3	0.00	0.00
Aristophania	0	0	1	2	0.00	0.00
Asaia	0	0	10	68	0.00	0.00
Bombella	0	0	8	23	0.00	0.00
Brytella	0	0	1	2	0.00	0.00
Commensalibacter	0	0	9	62	0.00	0.00
Endobacter	0	0	1	3	0.00	0.00
Entomobacter	0	0	1	1	0.00	0.00
Gluconacetobacter	0	0	28	36	0.00	0.00
Gluconobacter	0	0	28	148	0.00	0.00
Granulibacter	0	0	1	10	0.00	0.00
Komagataeibacter	1	1	19	83	5.26	1.20
Kozakia	0	0	1	6	0.00	0.00
Neoasaia	0	0	1	3	0.00	0.00
Neokomagataea	0	0	3	6	0.00	0.00
Nguyenibacter	0	0	1	3	0.00	0.00
Novacetimonas	1	2	4	25	25.00	8.00
Oecophyllibacter	0	0	1	3	0.00	0.00
Saccharibacter	0	0	1	8	0.00	0.00
Sorlinia	0	0	1	1	0.00	0.00
Swaminathania	0	0	1	2	0.00	0.00
Swingsia	0	0	1	1	0.00	0.00
Tanticharoenia	0	0	2	2	0.00	0.00
All	5	7	184	873	2.72	0.80

Table S3. Descriptive statistics of PRK00481 sirtuins.

Genus	Number of species with PRK00481 sirtuins	Number of genomes with PRK00481 sirtuins	Number of all species (child taxa)	Number of all genomes	Percentage of species with PRK00481 sirtuins (%)	Percentage of genomes with PRK00481 sirtuins (%)
Acetobacter	13	47	58	366	22.41	12.84
Acidomonas	0	0	1	6	0.00	0.00
Ameyamaea	1	2	1	3	100.00	66.67
Aristophania	0	0	1	2	0.00	0.00
Asaia	0	0	10	68	0.00	0.00
Bombella	0	0	8	23	0.00	0.00
Brytella	0	0	1	2	0.00	0.00
Commensalibacter	7	20	9	62	77.78	32.26
Endobacter	1	1	1	3	100.00	33.33
Entomobacter	1	1	1	1	100.00	100.00
Gluconacetobacter	12	19	28	36	42.86	52.78
Gluconobacter	0	0	28	148	0.00	0.00
Granulibacter	0	0	1	10	0.00	0.00
Komagataeibacter	15	43	19	83	78.95	51.81
Kozakia	0	0	1	6	0.00	0.00
Neoasaia	0	0	1	3	0.00	0.00
Neokomagataea	0	0	3	6	0.00	0.00
Nguyenibacter	2	6	1	3	200.00	200.00
Novacetimonas	4	9	4	25	100.00	36.00
Oecophyllibacter	0	0	1	3	0.00	0.00
Saccharibacter	0	0	1	8	0.00	0.00
Sorlinia	0	0	1	1	0.00	0.00
Swaminathania	0	0	1	2	0.00	0.00
Swingsia	0	0	1	1	0.00	0.00
Tanticharoenia	0	0	2	2	0.00	0.00
All	56	148	184	873	30.43	16.95

Table S4. Results of Fisher's exact test for number of species and number of genomes with SIR2 sirtuins.

χ^2 Tests		
	Value	p
Fisher's exact test		1.000
N	25	

Table S5. Results of Fisher's exact test for number of species and number of genomes with SIR2_2 sirtuins.

χ^2 Tests		
	Value	p
Fisher's exact test		1.000
N	25	

Table S6. Results of Fisher's exact test for number of species and number of genomes with PRK00481 sirtuins.

χ^2 Tests		
	Value	p
Fisher's exact test		1.000
N	25	

Table S7. Results of Kruskal-Wallis test for number of species and number of genomes with SIR2 sirtuins and for percentage of species and percentage of genomes with SIR2 sirtuins.

Kruskal-Wallis			
	χ^2	df	p
Number of species with SIR2 sirtuins	24.0	24	0.462
Number of genomes with SIR2 sirtuins	24.0	24	0.462
Percentage of species with SIR2 sirtuins	24.0	24	0.462
Percentage of genomes with SIR2 sirtuins	24.0	24	0.462

Table S8. Results of Kruskal-Wallis test for number of species and number of genomes with SIR2_2 sirtuins and for percentage of species and percentage of genomes with SIR2_2 sirtuins.

Kruskal-Wallis			
	χ^2	df	p
Number of species with SIR2_2 sirtuins	24.0	24	0.462
Number of genomes with SIR2_2 sirtuins	24.0	24	0.462
Percentage of species with SIR2_2 sirtuins	24.0	24	0.462
Percentage of genomes with SIR2_2 sirtuins	24.0	24	0.462

Table S9. Results of Kruskal-Wallis test for number of species and number of genomes with PRK00481 sirtuins and for percentage of species and percentage of genomes with PRK00481 sirtuins.

Kruskal-Wallis			
	χ^2	df	p
Number of species with PRK00481 sirtuins	24.0	24	0.462
Number of genomes with PRK00481 sirtuins	24.0	24	0.462
Percentage of species with PRK00481 sirtuins (%)	24.0	24	0.462
Percentage of genomes with PRK00481 sirtuins (%)	24.0	24	0.462

Table S10. Results of correlation analysis between percentage of species with SIR2 sirtuins and percentage of genomes with SIR2 sirtuins.

Correlation Matrix			
		Percentage of species with SIR2 sirtuins (%)	Percentage of genomes with SIR2 sirtuins (%)
Percentage of species with SIR2 sirtuins (%)	Spearman's rho	—	
	df	—	
	p-value	—	
Percentage of genomes with SIR2 sirtuins (%)	Spearman's rho	0.996	—
	df	23	—
	p-value	< .001	—

Table S11. Results of correlation analysis between percentage of species with SIR2_2 sirtuins and percentage of genomes with SIR2_2 sirtuins.

Correlation Matrix			
		Percentage of species with SIR2_2 sirtuins (%)	Percentage of genomes with SIR2_2 sirtuins (%)
Percentage of species with SIR2_2 sirtuins (%)	Spearman's rho	—	
	df	—	
	p-value	—	
Percentage of genomes with SIR2_2 sirtuins (%)	Spearman's rho	1.000	—
	df	23	—
	p-value	< .001	—

Table S12. Results of correlation analysis between percentage of species with PRK00481 sirtuins and percentage of genomes with PRK00481 sirtuins.

Correlation Matrix			
		Percentage of species with PRK00481 sirtuins (%)	Percentage of genomes with PRK00481 sirtuins (%)
Percentage of species with PRK00481 sirtuins (%)	Spearman's rho	—	
	df	—	
	p-value	—	
Percentage of genomes with PRK00481 sirtuins (%)	Spearman's rho	0.980	—
	df	23	—
	p-value	< .001	—

Table S13. Results of multinomial logistic regression for number of species with SIR2 sirtuins.

Model Fit Measures			
Model	Deviance	AIC	R^2McF
1	1.63e-4	200	1.000

Table S14. Results of multinomial logistic regression for number of genomes with SIR2 sirtuins.

Model Fit Measures			
Model	Deviance	AIC	R^2McF
1	1.68e-4	200	1.000

Table S15. Results of multinomial logistic regression for number of species with SIR2_2 sirtuins.

Model Fit Measures			
Model	Deviance	AIC	R^2McF
1	1.84e-4	100	1.000

Table S16. Results of multinomial logistic regression for number of genomes with SIR2_2 sirtuins.

Model Fit Measures			
Model	Deviance	AIC	R^2McF
1	1.54e-4	150	1.000

Table S17. Results of multinomial logistic regression for number of species with PRK00481 sirtuins.

Model Fit Measures			
Model	Deviance	AIC	R^2_{McF}
1	1.86e-4	350	1.000

Table S18. Results of multinomial logistic regression for number of genomes with PRK00481 sirtuins.

Model Fit Measures			
Model	Deviance	AIC	R^2_{McF}
1	1.10e-4	400	1.000

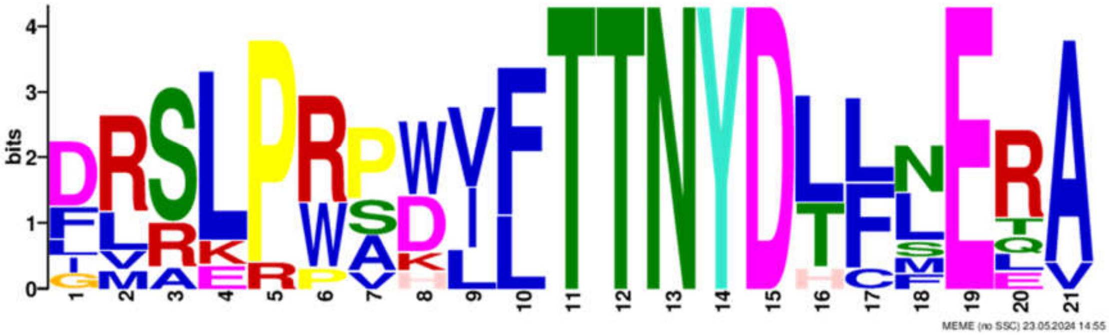

Figure S1. 9th motif of Figure 7.

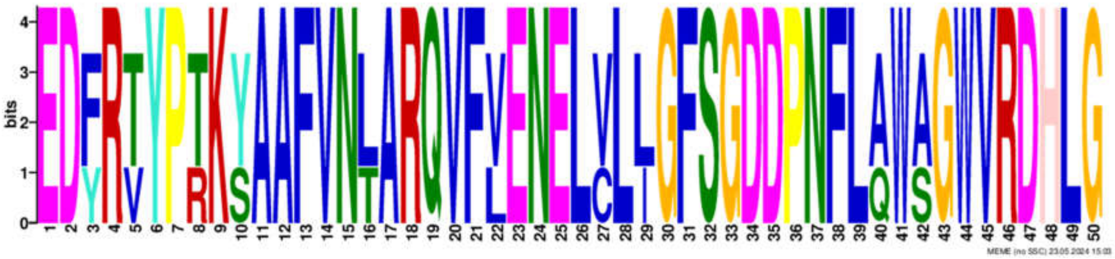

Figure S2. 13th motif of Figure 7.

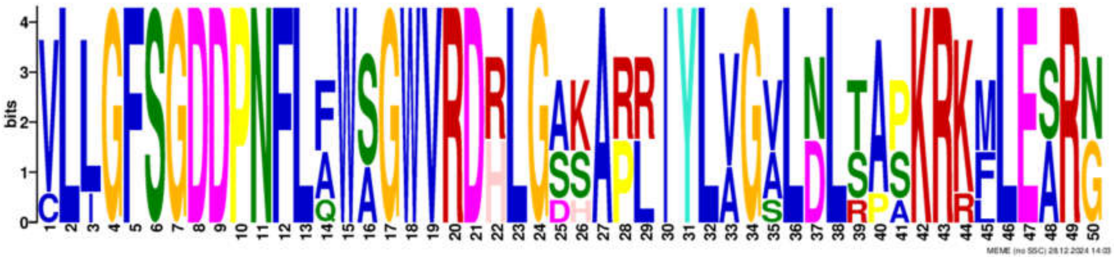

Figure S3. 9th motif of Figure 8.

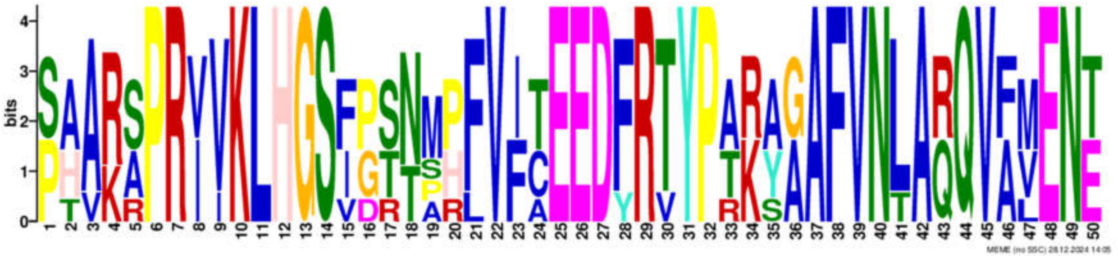

Figure S4. 16th motif of Figure 8.

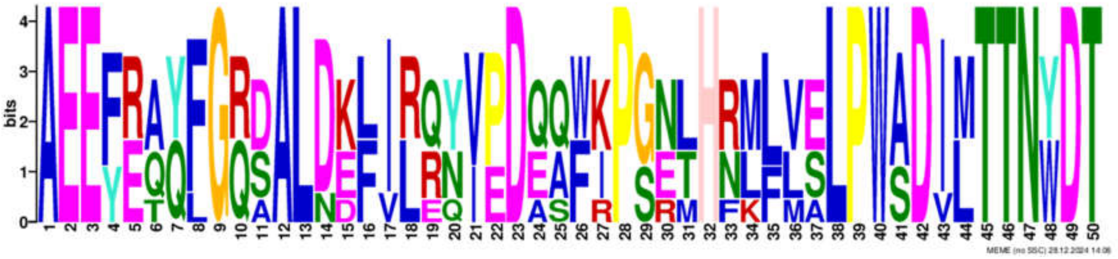

Figure S5. 17th motif of Figure 8.

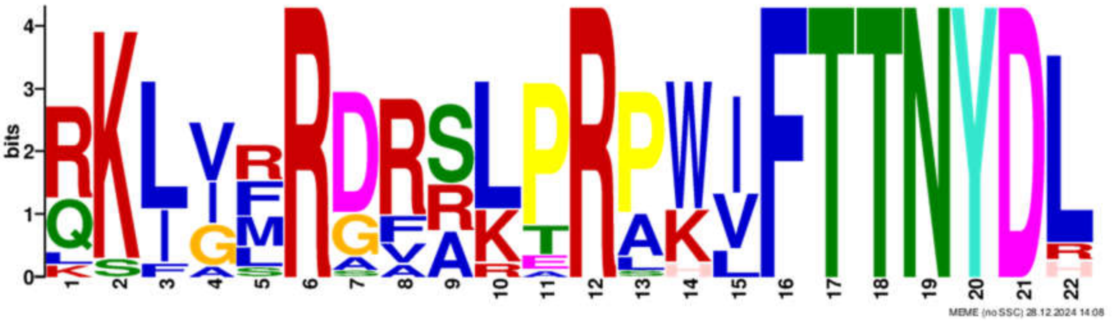

Figure S6. 3rd motif of Figure 8.

SIR2_2 Sirtuin Variant Detector of the Acetic Acid Bacteria

```
from Bio import SeqIO
from Bio.Seq import Seq
from Bio.SeqRecord import SeqRecord
from Bio.Align import PairwiseAligner

# Motifs to be checked
motifs = {
    9: "VLLGFSGDDPNFLFWSGWVRDRLGAKAPLIYLVGVLDLTAPKRKMLEARG",
    16: "PAARSPRVVKLHGSFPSNMPFVFTEEDFRTYPARAGAFVNLAQQVAMENT",
    17: "AEEFEAQFGRDALDKLILQYVPDQQFKPGNLHRMLVELPWADIMTTNWDT"
}

# Function to align sequences and detect similarity
def detect_sir2_2_variant(query_sequence, motif_threshold=0.9):
    aligner = PairwiseAligner()
    aligner.mode = 'global'

    motif_matches = []
    for motif_id, motif_seq in motifs.items():
        motif_alignment = aligner.align(query_sequence, motif_seq)
        motif_identity = motif_alignment.score / len(motif_seq)
        if motif_identity >= motif_threshold:
            motif_matches.append(motif_id)

    return motif_matches

# Get query sequence from console input
query_sequence = input("Enter the protein sequence to analyze: ")

motif_matches = detect_sir2_2_variant(query_sequence)
```

```
if len(motif_matches) == len(motifs):
    print("Query sequence matches all required motifs.")
    print(f"Motif Matches: {motif_matches}")
    print(f"This is a SIR2_2 sirtuin variant of the acetic acid bacteria.")
else:
    print("Query sequence does not match all required motifs.")
    print(f"Matched Motifs: {motif_matches}")
    print(f"This is not a SIR2_2 sirtuin variant of the acetic acid
bacteria.")
```
